# Visual hallucinations and age-related macular degeneration: case presentation and a brief literature review

**DOI:** 10.1192/j.eurpsy.2023.1982

**Published:** 2023-07-19

**Authors:** M. M. Gutiérrez Rodríguez, F. García Sánchez, M. Á. Corral Alonso, J. J. Vázquez Vázquez, C. Moreno Menguiano

**Affiliations:** Psychiatry, Hospital Universitario de Móstoles, Madrid, Spain

## Abstract

**Introduction:**

age-related macular degeneration (AMD) is an ocular disease involving central vision. It is one of the mainreasons of vision loss in people over 50. Seeing non-existing faces or shapes are described in AMD. Symtoms of visualhallucinations that occur as a result of vision loss is known as Charles Bonnet syndrome (CBS). These patients haveintact cognition, do not have hallucinations in any other sensory modalities, and retain insight into the unreal nature oftheir hallucinations.

**Objectives:**

the aim of this work is analizing ethiology, demographic characteristics, clinica features and treatment inpatients with AMD and visual hallucinations

**Methods:**

a literature search using electronic manuscripts available in PubMed database published during the last ten years with further description and discussion of a single-patient clinical case.

**Results:**

in different studies in patients diagnosed with AMD, the reported prevalence ranges between 15 up to 39percent. Patients with more significant vision loss may be more likely to experience visual hallucinations. In large caseseries, mean age is 70 to 85 years. Hallucinations can last few minutes or several hours. On average, people experiencethese hallucinations on and off for about 3 years. Those who experience hallucinations tend to see multiple types ofimages, particularly people and faces.The diagnosis of CBS is made when visual hallucinations occur in patients withvision loss in the absence of psychosis, delirium, or other causes.

There is no specific treatment for CBS: optimal ocular care, education and differents techniques to manage hallucinations(changing your lighting conditions and environment, blinking frequently or moving your eyes side-to-side rapidly whilekeeping your head still…). Antidepressants, anticonvulsants, anxiolytics and low-dose of antipsychotics have been used for CBS with positiveeffects in previous reports, but the efficacy of these drugs in the treatment is somewhat questionable and should bereserved for those who exhibit high levels of distress and have not responded to conventional intervention.

Case report: 80-years old woman who presented with a 4 month history of hallucinations and legally blind from AMD. Aworkup for other pathological causes of visual hallucinations was negative.

**Conclusions:**

CBS is an under-recognized and under-reported disorder that involves visual hallucinations in visuallyimpaired individuals. It requires a multidisciplinary approach from neurologists, psychiatrists, general practitioners andophthalmologists. New studies are needed in order to understand its clinical presentation and to improve its management.

**Disclosure of Interest:**

None Declared

